# Osteopathic manipulative treatment for nonspecific low back pain: a systematic review and meta-analysis

**DOI:** 10.1186/1471-2474-15-286

**Published:** 2014-08-30

**Authors:** Helge Franke, Jan-David Franke, Gary Fryer

**Affiliations:** INIOST-Institute for Osteopathic Studies, Siegen, Germany; College of Health & Biomedicine; ISEAL, Victoria University, Melbourne, Australia; A.T. Still Research Institute, A.T. Still University of Health Sciences, Kirksville, Missouri USA

**Keywords:** Low back pain, Spinal manipulation, Osteopathic manipulative treatment, Systematic review

## Abstract

**Background:**

Nonspecific back pain is common, disabling, and costly. Therefore, we assessed effectiveness of osteopathic manipulative treatment (OMT) in the management of nonspecific low back pain (LBP) regarding pain and functional status.

**Methods:**

A systematic literature search unrestricted by language was performed in October 2013 in electronic and ongoing trials databases. Searches of reference lists and personal communications identified additional studies. Only randomized clinical trials were included; specific back pain or single treatment techniques studies were excluded. Outcomes were pain and functional status. Studies were independently reviewed using a standardized form. The mean difference (MD) or standard mean difference (SMD) with 95% confidence intervals (CIs) and overall effect size were calculated at 3 months posttreatment. GRADE was used to assess quality of evidence.

**Results:**

We identified 307 studies. Thirty-one were evaluated and 16 excluded. Of the 15 studies reviewed, 10 investigated effectiveness of OMT for nonspecific LBP, 3 effect of OMT for LBP in pregnant women, and 2 effect of OMT for LBP in postpartum women. Twelve had a low risk of bias. Moderate-quality evidence suggested OMT had a significant effect on pain relief (MD, -12.91; 95% CI, -20.00 to -5.82) and functional status (SMD, -0.36; 95% CI, -0.58 to -0.14) in acute and chronic nonspecific LBP. In chronic nonspecific LBP, moderate-quality evidence suggested a significant difference in favour of OMT regarding pain (MD, -14.93; 95% CI, -25.18 to -4.68) and functional status (SMD, -0.32; 95% CI, -0.58 to -0.07). For nonspecific LBP in pregnancy, low-quality evidence suggested a significant difference in favour of OMT for pain (MD, -23.01; 95% CI, -44.13 to -1.88) and functional status (SMD, -0.80; 95% CI, -1.36 to -0.23), whereas moderate-quality evidence suggested a significant difference in favour of OMT for pain (MD, -41.85; 95% CI, -49.43 to -34.27) and functional status (SMD, -1.78; 95% CI, -2.21 to -1.35) in nonspecific LBP postpartum.

**Conclusion:**

Clinically relevant effects of OMT were found for reducing pain and improving functional status in patients with acute and chronic nonspecific LBP and for LBP in pregnant and postpartum women at 3 months posttreatment. However, larger, high-quality randomized controlled trials with robust comparison groups are recommended.

**Electronic supplementary material:**

The online version of this article (doi:10.1186/1471-2474-15-286) contains supplementary material, which is available to authorized users.

## Background

Low back pain (LBP) is defined as pain located below the costal margin and above the inferior gluteal folds[1]. Specific causes of LBP are uncommon, accounting for less than 15% of all back pain[2]. About 85% of patients with isolated LBP cannot be given a precise pathoanatomical diagnosis[3]. Nonspecific LBP has been defined as tension, soreness, and/or stiffness in the lower back region for which it is not possible to identify a specific cause of the pain[4]. It commonly leads to a loss of function and limitations in activities and participation in social life[5]. Because LBP pain is common in Western industrial societies, the economic consequences of back pain are enormous[6], and the effect on quality of life is substantial[7].

Back pain in pregnant and postpartum women is also common. The prevalence of LBP in pregnancy ranges from 24% to 90%, although it is most commonly estimated at 40%-50%[8–10]. Prevalence increases with the duration of pregnancy and is at the highest point in the third trimester[11, 12]. The cause of pain appears to be nonspecific and may be related to changes in body posture with the development of joint, ligament, and myofascial dysfunctions[13, 14]. The prevalence of LBP in postpartum women increases in the year after delivery, with estimates from 28% after 3 months to over 50% after 5 months and 67% after 12 months[15–18].

Osteopathy is a health approach that emphases the role of the musculoskeletal system in health and promotes optimal function of the tissues of the body by using a variety of manual techniques to improve the function of the body[19]. In the United States, practitioners are known as osteopathic physicians and have full medical licence. Osteopathic manipulative treatment (OMT) typically involves an eclectic range of manual techniques, which may include soft tissue stretching, spinal manipulation, resisted isometric ‘muscle energy’ stretches, visceral technique, or exercise prescription, for example. Treatment is characterised by a holistic approach to the patient, and OMT may be applied to many regions and tissues of the body, sometimes remote from the symptomatic area and at the clinical judgement of the practitioner[19–21].

Patients with LBP visit osteopaths for treatment, although the number of patients consulting osteopaths is not clear. In the United Kingdom, osteopaths were estimated to perform 4.38 million treatments in 1998[22]. Lumbar symptoms are the most common presentation in osteopathic practice in the United Kingdom, and, in a national pilot survey[23] and a snap-shot survey[24], accounted for 36% and 46% of presenting symptoms in patients, respectively. In Australia, the osteopathic profession is relatively small, and of those patients with LBP who chose to see a practitioner, medical practitioners (22.4%) and chiropractors (19.3%) are the most popular care providers, with only 2.7% of patients seeing osteopaths[25]. Despite this, LBP is the most common patient presentation in osteopathic practices[26].

In the United States, osteopathic physicians are more likely to provide LBP care than their allopathic medical counterparts[27].

To our knowledge, only 2 systematic reviews exist for osteopathic treatment of LBP. In 2005, Licciardone et al.[28] published the first systematic review of OMT for LBP and concluded that OMT significantly reduced LBP. This review had a number of limitations and was criticized because it did not differentiate between OMT and single manual techniques[29] and because single techniques do not reflect osteopathic clinical practice. Further, it combined dichotomous and continuous outcomes, combined studies with specific and nonspecific back pain, lacked a risk of bias evaluation, and contained a unit of analysis error. Given these shortcomings, reservations remain concerning the authors’ conclusions[29].

In 2012, Orrock and Myers[30] published another systematic review of OMT for LBP. This review only included studies of chronic nonspecific LBP and was limited to those published in English[30]. Only 2 studies met their specific search criteria, so no meta-analysis or robust conclusions were possible.

The objective of the current review was to examine the effectiveness of OMT for improving pain and functional status in LBP patients as compared to control treatments (no treatment, sham, and all other treatments) for adults in randomized clinical trials. Although 2 systematic reviews have been published on this topic, we were aware of recent, non-English studies that were not included in the previous reviews. Further, we intended to search the non-published ‘grey’ literature for studies which have not been published in journals or books, as recommended by the Cochrane Collaboration[31] for preparing and updating high-quality systematic reviews. We expected that using rigorous methodology and an extensive search without language restriction would provide a more comprehensive insight into the effectiveness of this intervention.

## Methods

### Criteria for considering studies for the current review

#### Types of studies

Only randomized controlled studies (RCTs) were included in the current review. Potential studies could be published or unpublished (grey literature) in any language.

#### Types of participants

We included studies with adults (older than 18 years) with nonspecific LBP (i.e., pain between the lumbo-pelvic region and the 12th rib) and without any limitation of the duration of the pain period (acute, subacute, or chronic back pain). We excluded studies which included participants with specific LBP (back pain with a specific cause, e.g., compression fracture, a tumour or metastasis, ankylosing spondylitis, infection).

There is a high prevalence of LBP associated with pregnancy and the postpartum period. Pregnancy and postpartum can be considered risk factors for nonspecific LBP, but they are not considered specific pathologies (e.g., infection, tumour, osteoporosis, ankylosing spondylitis, fracture, inflammatory conditions). Therefore, these groups can have specific or nonspecific LBP. For this reason we included studies that examined nonspecific LBP in pregnant and postpartum women, but presented these results as separate comparisons, even though other systematic reviews have excluded this subgroup without clear justification[32, 33].

#### Types of interventions

Treatment was required to be an ‘authentic’ OMT intervention where the practitioners were identified as osteopaths or osteopathic physicians and had a choice of manual techniques and judgment was required for the treatment selection, without any technique restrictions or standardised treatment protocols. The techniques chosen were based on the treating examiner’s opinion of what techniques would be most appropriate for a given patient. This eclectic, pragmatic approach best represents ‘real-world’ osteopathic practice[34–36], as opposed to treatment following an established study protocol that applies an isolated manual technique or set of techniques.

Therefore, our inclusion criteria were RCTs of OMT for nonspecific LBP where the treating practitioner was an osteopath or osteopathic physician who used clinical judgment to determine the treatment performed. Only studies where an effect size could be assigned to the OMT intervention were considered. If co-interventions were used, they also had to be performed in the control group. Studies were excluded that used an intervention of a single manual technique, such as high-velocity manipulation.

#### Types of comparisons

Studies with any type of comparison intervention (e.g., manual therapy, usual care, sham treatment, untreated) were included.

#### Types of outcome measures

Only patient-reported outcome measures were evaluated.

### Primary outcomes

The primary outcomes were pain and functional status. Pain was measured by visual analogue scale (VAS), number rating scale (NRS), or the McGill Pain Questionnaire. Studies measured functional status using the Roland-Morris Disability Questionnaire, Oswestry-Disability Index, or another valid instrument. For the meta-analysis, the outcome measure (pain or functional status) closest to the 3 month interval was used, even if studies used various time point measurements, because this interval was common for most of the included studies.

### Secondary outcome

These outcomes included any kind of adverse event.

### Data sources and searches

A systematic literature search was performed in October 2013 in the following electronic databases: Cochrane Central Register of Controlled Trials (CENTRAL), MEDLINE, Embase, CINAHL, PEDro, OSTMED.DR, and Osteopathic Web Research. The following search terms were used: low back pain, back pain, lumbopelvic pain, dorsalgia, osteopathic manipulative treatment, OMT, and osteopathic medicine. In addition to the listed databases, an ongoing trial database was also screened (metaRegister of Controlled Trialshttp://controlled-trials.com/mrct/). Our search was supplemented by citation tracking of the identified trials and a manual search in the reference lists for all relevant papers that were not listed in the electronic databases. Table 1 shows an example of the applied search strategy in MEDLINE.

**Table 1:** **Example search strategy**

Search terms and strategy used for MEDLINE	
1. randomized controlled trial.pt.	16. coccydynia.ti,ab.
2. controlled clinical trial.pt.	17. sciatica.ti,ab.
3. randomized.ab.	18. sciatic neuropathy
4. placebo.ab,ti.	19. spondylosis.ti,ab.
5. randomly.ti.ab.	20. lumbago.ti,ab.
6. trial.ab,ti.	21. low back pain.ti,ab.
7. groups.ab,ti.	22. lumbopelvic pain.ti,ab.
8. or/1-7	23. or/11-22
9. (animals not (humans and animals)).sh.	24. 10 and 23
10. 8 not 9	25. osteopathic medicine.mh.
11. dorsalgia.ti,ab.	26. manipulation, osteopathic.mh
12. back Pain. ti,ab	27. OMT.ti,ab.
13. backache.ti,ab.	28. or/25-27
14. (lumbar adj pain).ti,ab.	29. 24 and 28
15. coccyx.ti,ab.	

*Abbreviations*: *mh* Major heading, *pt* Publication type, *ti,ab* Title and Abstract.

### Data collection and analysis

#### Study selection

All authors independently screened titles and abstracts of the results identified by our search strategy. Potentially eligible studies were read in full text and independently evaluated for inclusion in the current review.

### Data extraction and quality assessment

The authors independently extracted data from identified studies using a standardized data extraction form.

### Dealing with missing data

If the article did not contain sufficient information, the authors were contacted for additional information. When standard deviations (SDs) were not reported, we estimated these from the confidence intervals (CIs) or other measures of variance, where possible. When the results were reported in median and interquartile range (IQR), we expected that the relation of median to mean was 1:1[37] and IQR to SD was 1.35:1[31]. If the normal distribution was skewed, we calculated the missing SD from the SDs of included studies that had similar results for outcome, comparison, and duration of pain[31].

### Assessment of heterogeneity

Heterogeneity refers to the variation in study outcomes between studies and is useful for the interpretation of meta-analysis results. Assessment of heterogeneity was based on the calculation of I^2^. The Cochrane Collaboration[31] provides the following interpretation of I^2^: 0% to 30%, might not be important; 30% to 60%, may represent moderate heterogeneity; 50% to 90%, may represent substantial heterogeneity; and 75% to 100%, considerable heterogeneity.

### Unit of analysis issues

In cases where 3 or more interventions were evaluated in a single study, we included each pair-wise comparison separately. In these instances, the total number of participants in the OMT intervention group was divided approximately evenly among the comparison groups.

### Assessment of risk of bias in included studies

The methodological quality of the studies was assessed using the Risk of Bias tool of the Cochrane Back Review Group[38]. Discussion and consensus between the researchers were used to resolve disagreements about the methodological quality of the RCTs included in the current review. Every Risk of Bias criterion was scored as ‘low risk’, ‘high risk’, or ‘unclear’ and included assessment of randomization, blinding, baseline comparability between groups, patient compliance, and dropping out. In line with recommendations from the Cochrane Back Review Group, studies were rated as having ‘low risk’ when at least 6 criteria were met and the study had no serious flaws (e.g., large drop-out rate). A dropout rate of greater than 50% was defined as a serious flaw and the comparison was excluded from quantitative analysis. When information was missing from the published studies and the authors could not be contacted or when the information was no longer available, the criteria were scored as ‘unclear’.

### Measures of treatment effect

Data for the meta-analysis was analysed using Review Manager (RevMan, Version 5.2., Nordic Cochrane Centre,http://ims.cochrane.org/revman). For measurement of pain, the NRS or VAS scores from the included studies were converted to a 100-point scale and the mean difference (MD) with 95% CIs was calculated in a random effects model. For functional status, the standard mean difference (SMD) was also used in a random effects model. Because only 1 study was included that examined acute LBP[39] and 3 other studies examined patients with both acute and chronic LBP[40–42], we grouped the studies into 4 groups for meta-analyses: acute and chronic LBP, chronic LBP (pain for a duration of greater than 3 months), LBP in pregnant women, and LBP in postpartum women.

### Assessment of clinical relevance

Assessment of clinical relevance was made using the recommendations of the Cochrane Back Review Group. Therefore, we defined a small effect as MD less than 10% of the scale (e.g., 10 mm on a 100 mm VAS) and SMD or ‘*d*’ scores less than 0.5. A medium effect was defined as MD 10% to 20% of the scale and SMD or ‘*d*’ scores from 0.5 to 0.8. A large effect was defined as MD greater than 20% of the scale and SMD or ‘*d*’ scores greater than 0.8[38].

### Data synthesis

The overall quality of the evidence for each outcome in the included studies was assessed using the GRADE approach[43, 44], as recommended by the updated Cochrane Back Review Group method guidelines[38]. The GRADE approach specifies 4 levels of quality, the highest rating being for RCT evidence. Authors of systematic reviews can downgrade this evidence to moderate, low, or even very low quality evidence, depending on the evaluation of quality of the evidence for each outcome against 5 key domains, which are (1) limitations in design (downgraded when more than 25% of the participants were from studies with a high Risk of Bias), (2) inconsistency of results (downgraded in the presence of significant statistical heterogeneity and inconsistent findings), (3) indirectness (i.e., generalisability of the findings, downgraded when more than 50% of the participants were outside the target group), (4) imprecision (downgraded when the total number of participants was less than 400 for each continuous outcome), and (5) other (such as publication bias)[33].

For the current review, the following definitions for quality of the evidence definitions were followed. For high quality, further research was very unlikely to change our confidence in the estimate of effect. There were also consistent findings among at least 75% of RCTs with no limitations of the study design and no known or suspected reporting biases. For moderate quality, further research was likely to have an important impact on confidence in the estimate of effect and may have changed the estimate; one of the domains was not met. For low quality, further research was very likely to have an important impact on confidence in the estimate of effect and was likely to change the estimate; 2 of the domains were not met. For very low quality, there was great uncertainty about the estimate; 3 of the domains were not met. For no evidence, no RCTs were identified that addressed the outcome. The research methods and reporting of this study adhered to the PRISMA guidelines[45].

## Results

### Included studies

**Figure 1:**
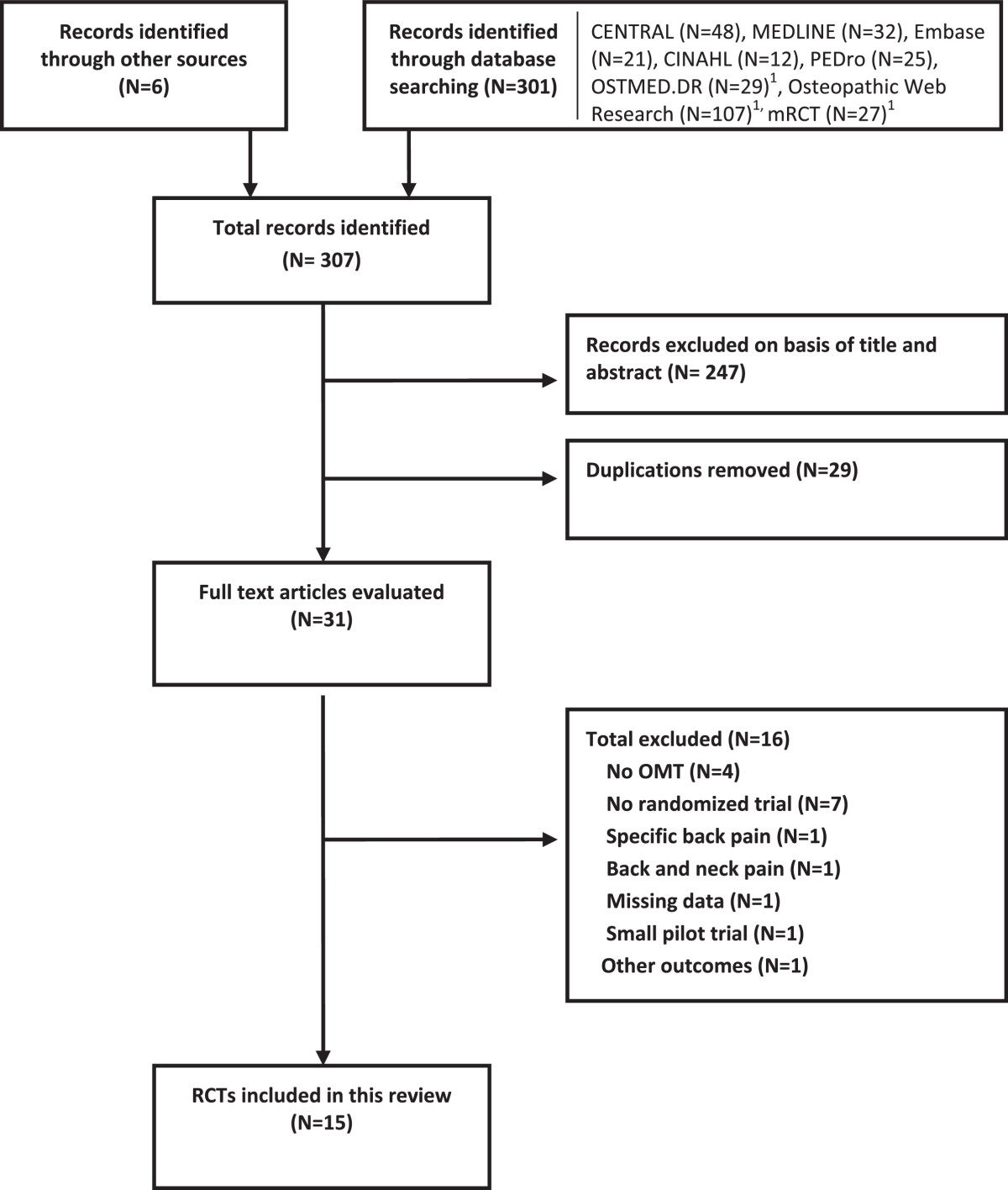
**Flowchart of study selection.** ^1^Sensitive and unspecific search, no adequate filter options possible. Abbreviations: mRCT, metaRegister of Controlled Trials; OMT, osteopathic manipulative treatment; RCT, randomized controlled trial.

The search strategy of the current review identified 307 studies (Figure 1). Fifteen trials[39–42, 46–56] with 18 comparison groups and 1502 participants were included in the qualitative and quantitative analysis. Tables 2 and3 summarize the important characteristics of the included studies. Of the 15 included studies, 6 were retrieved from the grey literature[42, 46, 48, 53–55]. Six studies came from Germany[42, 46, 48, 53–55], 5 from the United States[39, 40, 49–51], 2 from the United Kingdom[41, 47], and 2 from Italy[52, 56]. Ten studies investigated the effectiveness of OMT for back pain[39–42, 46, 47, 50–52, 56], 3 investigated the effect of OMT for LBP in pregnant women[48, 49, 53], and 2 investigated the effect of OMT for LBP in postpartum women[54, 55]. All included studies reported on pain and back pain-specific functional status, except for 1 study[41] that only reported pain.

**Table 2:** **Overview of treatment and comparison interventions in included studies**

Included study	Intervention	Comparison	Type of pain	Outcome measure interval^1^
Adorjàn-Schaumann 1999	OMT	Sham MT	Chronic	75 days
Andersson 1999	OMT	UC	Acute + Chronic	12 weeks
Chown 2008^3^	OMT	Physiotherapy	Chronic	6 weeks
Cruser 2012	OMT	UC	Acute	4 weeks
Gibson 1985	OMT	Sham SWD	Acute + Chronic^4^	2, 4, **12** weeks
Gibson 1985a^2^	OMT	SWD	Acute + Chronic^4^	2, 4, **12** weeks
Gundermann 2013	OMT	Untreated	NS, Pregnancy	6 weeks
Heinze 2006	OMT + PT + Heat	PT + Heat	Acute + Chronic	12 weeks
Licciardone 2003	OMT	Untreated	Chronic	30, **90**, 180 days
Licciardone 2003a^2^	OMT	Sham MT	Chronic	30, **90**, 180 days
Licciardone 2009	OMT + UOBC	UOBC	NS, Pregnancy	9 weeks
Licciardone 2009^2^	OMT + UOBC	UOBC + SUT	NS, Pregnancy	9 weeks
Licciardone 2013	OMT	Sham OMT	Chronic	12 weeks
Mandara 2008	OMT	Sham MT	Chronic	6 weeks
Peters 2006	OMT	Untreated	NS, Pregnancy	5 weeks
Recknagel 2007	OMT	Untreated	Chronic, PP	8 weeks
Schwerla 2012	OMT	Untreated	Chronic, PP	8 weeks
Vismara 2012	OMT + SE	SE	Chronic	2 weeks

^1^Bolded time interval included in the analysis of the current review.

^2^Second comparison in this published study.

^3^The second comparison in this study (group exercise) was not considered because of a dropout rate of 60%.

^4^Low back pain period from 2 weeks to 30 weeks.

*Abbreviations*: *MT* Manipulative treatment, *NS* Not specified, *OMT* Osteopathic manipulative treatment, *PP* Postpartum, *PT* Physical therapy, *SE* Specific exercises, *SUT* Sham ultrasound treatment, *SWD* Short-wave diathermy, *UC* Usual care, *UOBC* Usual obstetric care.

**Table 3:** **Overview of included randomised clinical trials of osteopathic manipulative treatment for low back pain**

Author/Year	Adorjàn-Schaumann 1999	Andersson 1999	Chown 2008	Cruser 2012	Gibson 1985
Country	Germany	United States	United Kingdom	United States	United Kingdom
**Aim of the study**	Can OMT provide a specified effect on the functional impairment and pain of patients with chronic lumbar back pain?	Comparison of OMT with standard care for patients with low back pain.	Is one to one physiotherapy or physiotherapy-led group exercise as effective as one to one osteopathy for patients with chronic low back pain?	Examination of efficacy of OMT in relieving acute low back pain and improving functioning in military personnel.	Comparison of OMT with SWD and placebo SWD in nonspecific low back pain.
**Duration of pain**	At least 6 months	At least 3 weeks, but less than 6 months	More than 3 months	Acute = minimum of 30 days hiatus of pain from previous LBP episodes	At least 2 months, but less than 12 months
**Reported inclusion/exclusion criteria**	Yes/Yes	Yes/Yes	Yes/Yes	Yes/Yes	Yes/Yes
**Outcome Measurement**	1. Roland Morris life quality score, 2. VAS, 3. SF-36 (modified), 4. Side effects	1. VAS, 2. RMDQ, 3. OPQ, 4. ROM, 5. Straight-leg raising	1. ODI, 2. EuroQol EQ-5D, 3. VAS, 4. Shuttle walk test	1. QVAS, 2. RMDQ, 3. SF-36, 4. Patient expectation questionnaire	1. VAS (daytime and nocturnal scores), 2. Spinal flexion, 3. Return to work, 4. Recovery, 5. Analgesic consumption
**No. of patients/dropouts**	57/10	178/23	239/85^4^	60/3	109/4^1^/ 5^2^/12^3^
**No. of patients/mean age**					
**a. Intervention**	a = 29/40.4 years	a = 83/28.5 years	a = 79/43.5 years	a = 30/26.3 years	a = 41/34 years
**b. Control**	b = 28/41.8 years	b = 72/37.0 years	b = 80/44.3 years	b = 30/27.1 years	b = 34/35 years
**c. Control**			c = 80/42.5 years		c = 34/40 years
**Treatment (No.)**					
**a. Intervention**	a = OMT (5)	a = OMT (8)	a = OMT (5)	a = OMT (4) + usual care	a = OMT (4)
**b. Control**	b = Sham treatment (5)	b = Standard medical therapies (8)	b = Physiotherapy (5)	b = Usual care	b = SWD (12)
**c. Control**			c = Group exercise (5)		c = Placebo SWD (12)
**Period**	60 days	12 weeks	3 months	4 weeks	4 weeks
**Authors’ conclusion**	‘OMT – in comparison to the sham treatment - shows statistically significant and clinically important improvements regarding primary and secondary outcome measures.’	‘Osteopathic manual care and standard medical care have similar clinical results in patients with subacute low back pain. However, the use of medication is greater with standard care.’	‘All three treatments indicated comparable reductions in mean (95% CI) ODI at 6-week follow-up....One-to-one therapies provided evidence of greater patient satisfaction.’	‘The study supports the effectiveness of OMT in reducing acute LBP pain in active duty military personnel.’	‘These observations indicate that neither osteopathic manipulation nor SWD was superior to placebo treatment.’
**Author/Year**	**Gundermann 2013**	**Heinze 2006**	**Licciardone 2003**	**Licciardone 2009**	**Licciardone 2013**
**Country**	**Germany**	**Germany**	**United States**	**United States**	**United States**
**Aim of the study**	To evaluate the effectiveness of osteopathic treatment in pregnant women suffering from LBP.	Determination of the efficacy of OMT applied to subacute lumbar back pain.	Determination of the efficacy of OMT as a complementary treatment for chronic nonspecific LBP.	Examination of OMT for back pain and related symptoms during the third trimester of pregnancy.	To study the efficacy of OMT and UST for chronic low back pain.
**Duration of pain**	At least 1 week	Between 4 weeks and 6 months	At least 3 months	Not specified	At least 3 months
**Reported inclusion/exclusion criteria**	Yes/Yes	Yes/Yes	Yes/Yes	Yes/Yes	Yes/Yes
**Outcome Measurement**	1. VAS, 2.Frequency of pain, 3. RMDQ, 4. Questionnaire (postpartum).	1. NRS for current and average level of pain, 2. RMDQ	1. SF-36, 2. VAS, 3. RMDQ, 4. Work disability, 5.Satisfaction with back care	1. Back pain on an 11-point scale, analysed like a 10-cm VAS for pain, 2. RMDC	1. VAS, 2. RMDQ, 3. SF-36 general health score, 4. Lost work days, 5.Satisfaction with back care, 5. Co-treatments.
**No. of patients/ Dropouts**	41/2	60/2	91/25	146/2 (Prior first visit)	455/93
**No. of patients/mean age**					
**a. Intervention**	a = 21/29 years	a = 28/42.1 years	a = 48/49 years	a = 49/23.8 years	a = 230/41 (median) years
**b. Control**	b = 20/31 years	b = 32/44.3 years	b = 23/52 years	b = 48/23.7 years	b = 225/40
**c. Control**			c = 20/49 years	c = 49/23.8 years	(median) years
**Treatment (No.)**					
**a. Intervention**	a = OMT (4)	a = OMT (2–3) + heat & PT (6)	a = OMT (7) + UC	a = UOBC + OMT (7)	a = OMT ^5^ (6)
**b. Control**	b = Untreated	b = Heat & PT (6)	b = Sham manipulation (7) + UC	b = UOBC + SUT (7)	b = Sham OMT ^5^ (6)
**c. Control/Period**			c = UC	c = UOBC	
	7 weeks	6 weeks	5 months	10 weeks	8 weeks
**Authors’ conclusion**	‘Four osteopathic treatments over a period of 8 weeks led to statistically significant and clinically relevant positive changes of pain intensity and frequency in pregnant women suffering from low back pain.’	‘In the area of pain, as well as in the area of the disabilities a clinically relevant improvement could be achieved.’	OMT and sham manipulation ‘both appear to provide some benefits when used in addition to usual care for the treatment of chronic nonspecific low back pain’.	‘Osteopathic manipulative treatment slows or halts the deterioration of back-specific functioning during the third trimester of pregnancy’.	‘The OMT regimen met or exceeded the Cochrane Back Review Group criterion for a medium effect size in relieving chronic low back pain. It was safe, parsimonious, and well accepted by patients.’
**Author/Year**	**Mandara 2008**	**Peters 2006**	**Recknagel 2007**	**Schwerla 2012**	**Vismara 2012**
**Country**	**Italy**	**Germany**	**Germany**	**Germany**	**Italy**
**Aim of the study**	To compare the effects of OMT with sham manipulative treatment (SMT) on patient’s self-reported pain and disability.	Assessment whether OMT influences the pain-symptomatology of women with pregnancy related low back pain.	Investigation whether OMT had an effect on women with post-partum persistent unspecific backache.	To evaluate the effectiveness of osteopathic treatment in women suffering from persistent low back pain after childbirth.	Is OMT combined with specific exercises more effective than specific exercises alone in obese female patients with chronic low back pain?
**Duration of pain**	More than 3 month	At least 1 week	At least 3 months, not more than 24 months	After childbirth for at least 3 months and at most 20 months	More than 6 months
**Reported inclusion/exclusion criteria**	No/No	Yes/Yes	Yes/Yes	Yes/Yes	Yes/Yes
**Outcome Measurement**	1. VAS, 2. ODI	1. VAS, 2. Quebec Back Pain disability scale	1. VAS, 2. OPQ, 3. Regions of dysfunction	1. VAS, 2. OPQ. 3. Different specific health problems	1. Kinematic of thoracic/ lumbar spine/pelvis during forward flexion, 2. VAS, 3. RMDQ, 4. LBP-DQ
**No. of patients/Dropouts**	94/6	60/3	40/1	80/3	21/2
**No. of patients/mean age**					
**a. Intervention**	a = 44/NS	a = 30/30.6 years	a = 20/34.5 years	a = 39/33.9 years	a = 8/42.0 years
**b. Control**	b = 50/NS	b = 30/30.2 years	b = 19/34.4 years	b = 40/33.3 years	b = 11/44.7 years
**c. Control**					
**Treatment (No.)**					
**a. Intervention**	a = OMT + Usual care (6)	a = OMT (4)	a = OMT (4)	a = OMT (4)	a = OMT (1) + SE (10)
**b. Control**	b = SMT + Usual care (6)	b = No treatment	b = No treatment	b = Untreated	b = SE (10)
**c. Control/Period**	6 weeks	4 weeks	8 weeks	8 weeks	NS
**Authors’ conclusion**	‘…OMT appears to provide benefits over and above usual care for the treatment of CLBP. The improvement in the OMT compared to the SMT demonstrated that placebo effects… do not justify per se the results of this study.’	‘Four osteopathic treatments… could cause a clinically relevant influence on the pain-symptomatology and on the interference of daily life of pregnant women with pain in the pelvic and/or lumbar area’.	OMT ‘for women with persistent, unspecific backache post-partum brings about a clinically relevant improvement of the pain symptoms and a reduction of the impediment on daily life’.	‘Four osteopathic treatments over a period of eight weeks led to statistically significant and clinically relevant positive changes of pain intensity and effects of low back pain on everyday activities in women suffering from low back pain after childbirth’	‘OMT + SE showed to be effective in improving biomechanical parameters of the thoracic spine in obese patients with chronic LBP …’

^1^ = After 2 weeks.

^2^ = After 4 weeks.

^3^ = After 12 weeks.

^4^ Dropouts intervention group = 16, Control physiotherapy = 21, Control group exercise = 48.

^5^ Main effect groups.

*Abbreviations*: *CI* Confidence interval, *LBP* Low back pain, *LBP-DQ* Low back pain disability questionnaire, *NRS* Numeric rating scale, *NS* Not specified, *OMT* Osteopathic manipulative treatment, *ODI* Oswestry Disability Index, *OPQ* Oswestry Pain Questionnaire, *PT* Physical therapy, *QVAS* Quadruple Visual Analogue Scale, *SUT* Sham ultrasound treatment, *RMDQ* Roland Morris Disability Questionnaire, *ROM* Range of motion, *SWD* Short-wave diathermy, *UC* Usual care, *UOBC* Usual obstetric care, *UST* Ultrasound treatment, *VAS* Visual analogue scale pain.

### Excluded studies

Sixteen of the 31 identified studies were excluded from our review (Figure 1). In 3 studies, the treatment involved only a single technique[57–59], and in 1 study the treatment was based on local fascial manipulations[60]. Seven studies did not use RCT methodology[61–63] (Conrady A, Döring R: Does osteopathic treatment influence immune parameters in patients with chronic low back pain? A pre-post pilot trial, unpublished D.O. thesis, Akademie für Osteopathie, 2010; Kofler G: Osteopathy for back and pelvic pain in pregnancy, unpublished D.O. thesis, Wiener Schule für Osteopathie, 2006; Lutzelberger N: Does osteopathic treatment influence thoracolumbar junction back pain positively? unpublished D.O. thesis, Akademie für Osteopathie, 2009; Müller P: Nonspecific, pseudoradicular low back pain after lumbar nucleotomy, unpublished D.O. thesis, Akademie für Osteopathie, 2006), and 1 study focused on specific LBP[5]. In another study, we could not differentiate the back pain results from the neck pain results[64]. One study used a non-validated disability index and did not report pain scores[65]. Another study focused on outcomes other than pain and functional status[66]. One pilot study was excluded because it focused only on feasibility[67].

### Risk of bias

Thirteen of the included studies in the meta-analysis had high internal validity (low risk of bias) (Table 4). The study by Licciardone et al.[51] from 2003 and Gibson[41] were found to have a high risk of bias, with both studies having only 5 criteria each assessed as low risk. Additionally, the second comparison group (OMT/group exercise) in the study by Chown et al.[47] was rated as having a high risk of bias because only 40% of the participants in the exercise group completed all therapy sessions. This comparison was excluded. In the 2009 study by Licciardone et al.[49], 83 of 144 participants withdrew before the last treatment. Although we determined this study had a high risk of bias, we included it in our analysis because the reasons for the dropouts were described and an intention-to-treat-analysis (last observation carried forward) was performed.

**Table 4:** **Risk of bias in the included studies**

	Randomisation?	Allocation concealed?	Patient blinding?	Care provider blinding?^1^	Outcome assessor blinding?^2^	Drop-outs described + acceptable?	Free of selective outcome report?	Groups similar at baseline?	Co-intervention avoided or similar?	Compliance acceptable?	Intention-to-treat analysis?	Similar timing outcome assessment?
**Study**	**1**	**2**	**3**	**4**	**5**	**6**	**7**	**8**	**9**	**10**	**11**	**12**
Adorjàn-Schaumann 1999	LR	LR	UC	HR	UC	LR	LR	LR	LR	HR	LR	LR
Andersson 1999	LR	LR	HR	HR	HR	LR	LR	LR	LR	LR	HR	LR
Chown 2008	LR	LR	UC	HR	UC	LR ^3^	LR	LR	LR	LR	UC	LR
Cruser 2012	LR	LR	HR	HR	HR	LR	LR	LR	LR	LR	LR	LR
Gibson 1985	UC	UC	HR	HR	HR	LR	LR	HR	LR	LR	UC	LR
Gundermann 2013	LR	LR	HR	HR	HR	LR	LR	LR	LR	LR	LR	HR
Heinze 2006	LR	LR	HR	HR	HR	LR	LR	HR	LR	LR	LR	LR
Licciardone 2003	LR	LR	UC	HR	UC	UC	HR	LR	LR	UC	UC	LR
Licciardone 2009	LR	UC	HR	HR	HR	HR	LR	LR	LR	LR	LR	LR
Licciardone 2013	LR	LR	UC	HR	UC	LR	HR	LR	LR	LR	LR	LR
Mandara 2008	LR	LR	UC	HR	UC	LR	LR	UC	LR	LR	HR	LR
Recknagel 2007	LR	LR	HR	HR	HR	LR	LR	HR	LR	LR	LR	LR
Peters 2006	LR	LR	HR	HR	HR	LR	LR	LR	LR	LR	HR	LR
Schwerla 2012	LR	LR	HR	HR	HR	LR	LR	HR	LR	LR	LR	LR
Vismara 2012	LR	LR	HR	HR	HR	LR	LR	LR	LR	LR	HR	LR

^1^In manual therapy studies, blinding is not possible.

^2^For patient-reported outcomes, a low risk of bias is only possible if there is a low risk of bias for participant blinding.

^3^Comparison between osteopathic manipulative treatment and physiotherapy group is low risk, but comparison between osteopathic manipulative treatment and exercise group is high risk (due to high dropout rate). This comparison was therefore excluded from the review.

*Abbreviations*: *HR* High risk of bias, *LR* Low risk of bias, *UC* Unclear.

### Effect of interventions

**Figure 2:**
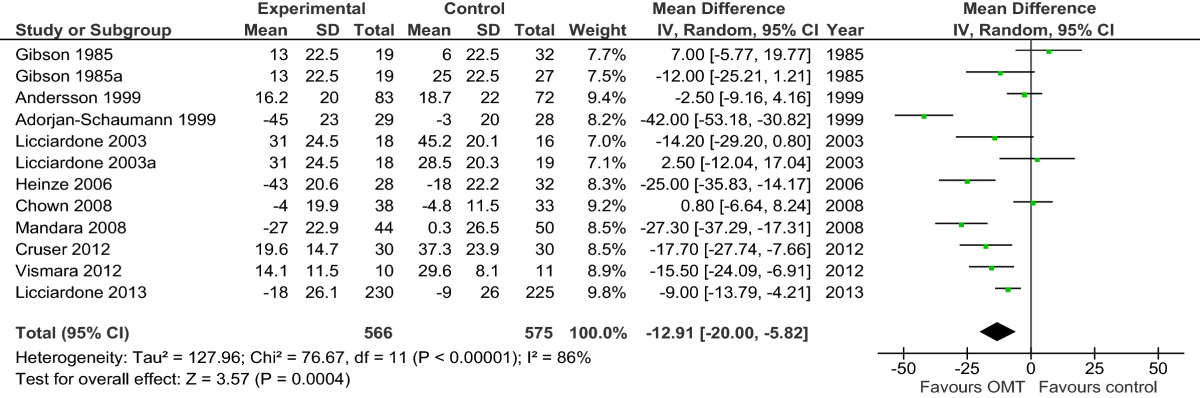
**Forest plot of comparison: OMT for low back pain – acute and chronic.** Outcome: pain. Abbreviations: CI, confidence interval; OMT, osteopathic manipulative treatment; SD, standard deviation.

**Figure 3:**
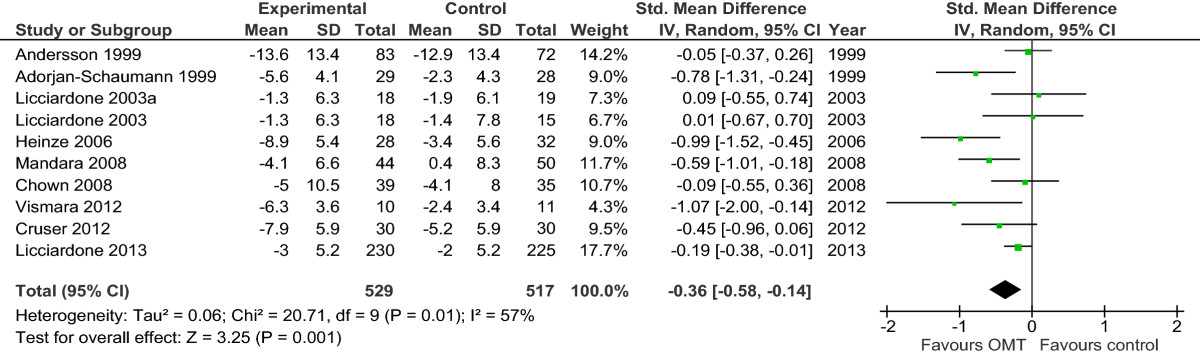
**Forest plot of comparison: OMT for low back pain – acute and chronic.** Outcome: functional status. Abbreviations: CI, confidence interval; OMT, osteopathic manipulative treatment; SD, standard deviation.

**Figure 4:**
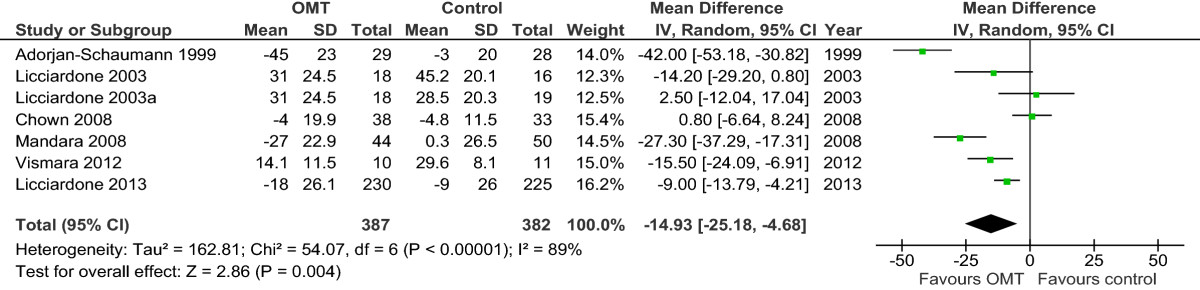
**Forest plot of comparison: OMT for low back pain – chronic.** Outcome: pain. Abbreviations: CI, confidence interval; OMT, osteopathic manipulative treatment; SD, standard deviation.

**Figure 5:**
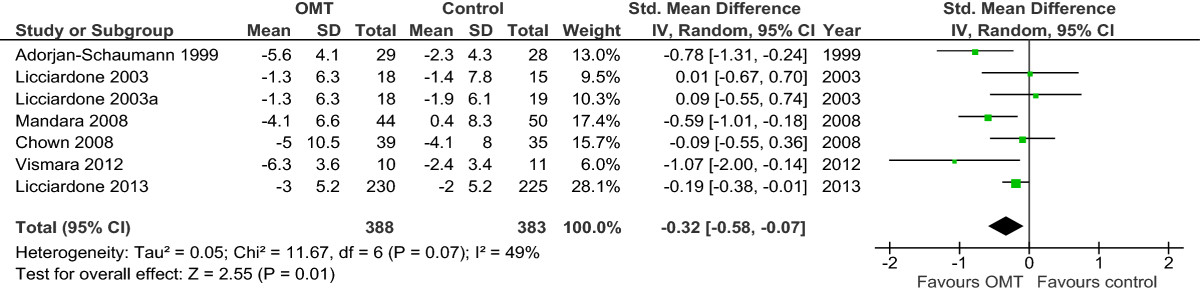
**Forest plot of comparison: OMT for low back pain – chronic.** Outcome: functional status. Abbreviations: CI, confidence interval; OMT, osteopathic manipulative treatment; SD, standard deviation.

**Figure 6:**
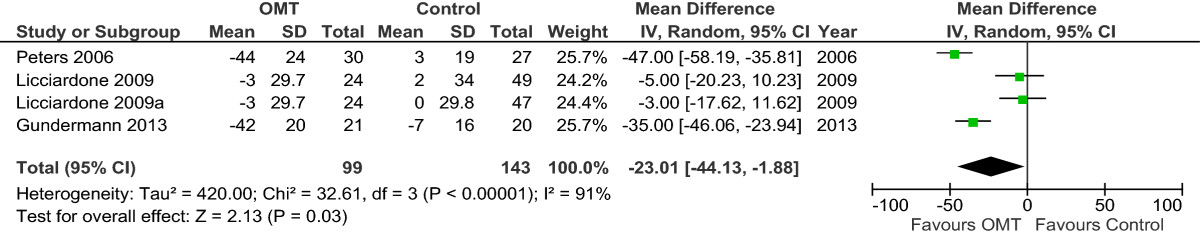
**Forest plot of comparison: OMT for low back pain – pregnancy.** Outcome: pain. Abbreviations: CI, confidence interval; OMT, osteopathic manipulative treatment; SD, standard deviation.

**Figure 7:**
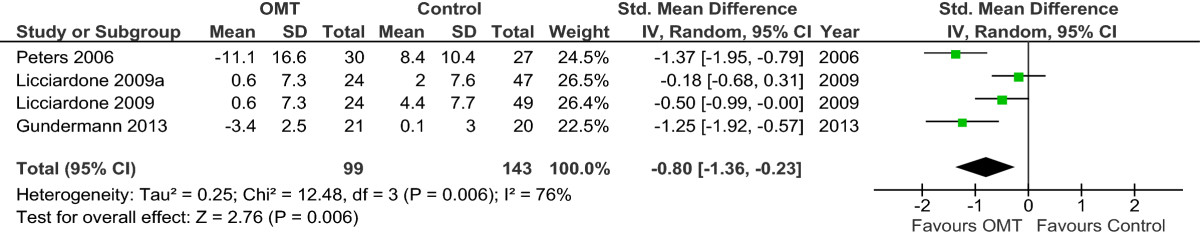
**Forest plot of comparison: OMT for low back pain – pregnancy.** Outcome: functional status. Abbreviations: CI, confidence interval; OMT, osteopathic manipulative treatment; SD, standard deviation.

**Figure 8:**
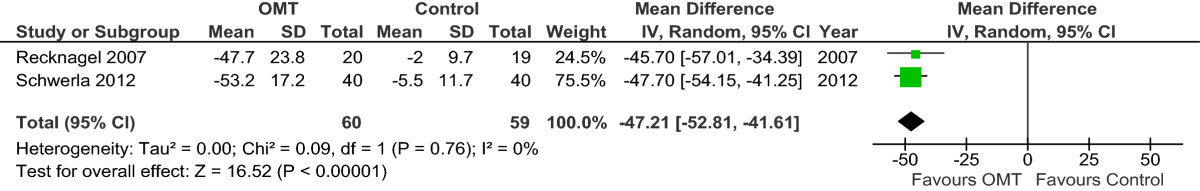
**Forest plot of comparison: OMT for low back pain – postpartum.** Outcome: pain. Abbreviations: CI, confidence interval; OMT, osteopathic manipulative treatment; SD, standard deviation.

Results are presented in the forest plots (Figures 2,3,4,5,6,7,8, and9) and in the summary of findings tables (Tables 5,6,7, and8). All results are based on measures which were closest to 3 months posttreatment.

**Figure 9:**
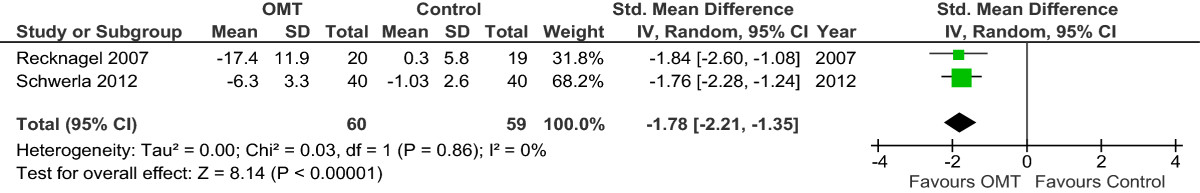
**Forest plot of comparison: OMT for low back pain – postpartum.** Outcome: functional status. Abbreviations: CI, confidence interval; OMT, osteopathic manipulative treatment; SD, standard deviation.

**Table 5:** **Osteopathic manipulative treatment in comparison to other interventions for acute and chronic nonspecific low back pain**

Quality assessment							No. of patients		Treatment effect (95% CI)	Quality of the evidence
No. of studies	Design	Limitations	Inconsistency	Indirectness	Imprecision	Other considerations	OMT	All other interventions and sham treatment		
**Pain (follow-up 3 months; measured with: Pain VAS from 0 to 100 [worse pain]; Better indicated by lower values)**										
10	randomised trials	no serious limitations	serious^1^	no serious indirectness	no serious imprecision	none	566	575	MD 12.91 lower (20 to 5.82 lower)	⊕ ⊕ ⊕Ο MODERATE
**Functional status (follow-up 3 months; measured with: Roland-Morris Disability Questionnaire, Oswestry Disability Index; Better indicated by lower values)**										
9	randomised trials	no serious limitations	serious^1^	no serious indirectness	no serious imprecision	none	529	517	SMD 0.36 lower (0.58 to 0.14 lower)	⊕ ⊕ ⊕Ο MODERATE

^1^I2 = 86%.

*Abbreviations*: *CI* Confidence interval, *MD* Mean difference, *OMT* Osteopathic manipulative treatment, *SMD* Standard mean difference, *VAS* Visual analogue scale.

**Table 6:** **Osteopathic manipulative treatment in comparison to other interventions for chronic nonspecific low back pain**

Quality assessment							No. of patients		Treatment effect (95% CI)	Quality of the evidence
No. of studies	Design	Limitations	Inconsistency	Indirectness	Imprecision	Other considerations	OMT	All other interventions and sham treatment		
**Pain (measured with: Pain VAS from 0 to 100 [worse pain]; Better indicated by lower values)**										
6	randomised trials	no serious limitations	serious^1^	no serious indirectness	no serious imprecision	none	387	382	MD 14.93 lower (25.18 to 4.68 lower)	⊕ ⊕ ⊕Ο MODERATE
**Functional status (follow-up 3 months; measured with: Roland-Morris Disability Questionnaire, Oswestry Disability Index; Better indicated by lower values)**										
6	randomised trials	no serious limitations	no serious inconsistency	no serious indirectness	no serious imprecision	none	388	383	SMD 0.32 lower (0.58 to 0.07 lower)	⊕ ⊕ ⊕ ⊕ HIGH

^1^I2 = 89%.

*Abbreviations*: *CI* Confidence interval, *MD* Mean difference, *OMT* Osteopathic manipulative treatment, *SMD* Standard mean difference, *VAS* Visual analogue scale.

**Table 7:** **Osteopathic manipulative treatment in comparison to usual obstetric care, sham ultrasound and untreated for nonspecific low back pain in pregnant women**

Quality assessment							No. of patients		Treatment effect (95% CI)	Quality of the evidence
No. of studies	Design	Limitations	Inconsistency	Indirectness	Imprecision	Other considerations	OMT	Usual obstetric care, sham ultrasound and untreated		
**Pain (measured with: Pain VAS from 0 to 100 [worse pain]; Better indicated by lower values)**										
3	randomised trials	no serious limitations	serious^1^	no serious indirectness	serious^2^	none	99	143	MD 23.01 lower (44.13 to 1.88 lower)	⊕ ⊕ ΟΟ LOW
**Functional status (measured with: Roland-Morris Disability Questionnaire, Quebec Back Pain Disability Scale; Better indicated by lower values)**										
3	randomised trials	no serious limitations	serious^3^	no serious indirectness	serious^2^	none	99	143	SMD 0.80 lower (1.36 to 0.23 lower)	⊕ ⊕ ΟΟ LOW

^1^I2 = 91%.

^2^Sample size < 400.

^3^I2 = 76%.

*Abbreviations*: *CI* Confidence interval, *MD* Mean difference, *OMT* Osteopathic manipulative treatment, *SMD* Standard mean difference, *VAS* Visual analogue scale.

**Table 8:** **Osteopathic manipulative treatment in comparison to untreated for nonspecific low back pain in postpartum women**

Quality assessment							No. of patients		Treatment effect (95% CI)	Quality of the evidence
No. of studies	Design	Limitations	Inconsistency	Indirectness	Imprecision	Other considerations	OMT	Untreated		
**Pain (measured with: Pain VAS from 0 to 100 [worse pain]; Better indicated by lower values)**										
2	randomised trials	no serious limitations	no serious inconsistency	no serious indirectness	serious^1^	none	60	59	MD 41.85 lower (49.43 to 34.27 lower)	⊕ ⊕ ⊕Ο MODERATE
**Functional status (measured with: Oswestry Pain Questionnaire; Better indicated by lower values)**										
2	randomised trials	no serious limitations	no serious inconsistency	no serious indirectness	serious^1^	none	60	59	SMD 1.78 lower (2.21 to 1.35 lower)	⊕ ⊕ ⊕Ο MODERATE

^1^Sample size < 400.

*Abbreviations*: *CI* Confidence interval, *MD* Mean difference, *OMT* Osteopathic manipulative treatment, *SMD* Standard mean difference, *VAS* Visual analogue scale.

### OMT versus other interventions for acute and chronic nonspecific low back pain

Ten studies with 12 comparison groups and 1141 participants were analysed for the effect of OMT for pain in acute and chronic LBP. Six studies reported a significant effect on pain in favour of OMT[39, 42, 46, 50, 52, 56], 3 studies reported a non-significant effect in favour of OMT[40, 41, 51], and 3 studies reported a non-significant effect in favour of the control treatment[41, 47, 51]. For pain, there was moderate-quality evidence (downgraded due to inconsistency) that OMT had a significant effect on pain relief (MD, -12.91; 95% CI, -20.00 to -5.82) (Figure 2 and Table 5).

For functional status, which was based on 9 studies with 10 comparisons and 1046 participants, there was moderate-quality evidence (downgraded due to inconsistency) of a significant difference in favour of OMT (SMD, -0.36; 95% CI, -0.58 to -0.14). Four studies reported a significant effect in favour of OMT[42, 46, 52, 56], 3 studies reported a non-significant effect in favour of OMT[39, 40, 47], and 1 study reported a non-significant effect in favour of the control group[51]. For 1 study[50], we estimated the effect size with a confidence interval which was very near to 0 (SMD, -0.19; 95% CI, -0.38 to -0.01) and significant (P = .04), whereas the authors reported that the results were not significant (P = .07, based on median and interquartile range) (Figure 3 and Table 5).

### OMT versus other interventions for chronic nonspecific low back pain

For the outcome of pain and based upon 6 studies[46, 47, 50–52, 56] with 7 comparisons and 769 participants, there was moderate-quality evidence (downgraded due to inconsistency) of a significant difference in favour of OMT (MD, -14.93; 95% CI, -25.18 to -4.68) (Figure 4 and Table 6).

For functional status, 3 studies reported a significant improvement for OMT[46, 52, 56], 1 reported a non-significant effect for OMT[47], and 1 reported an effect for the control group[51] (Figure 5). There was moderate-quality evidence (downgraded due to inconsistency) for a significant difference in favour of OMT (SMD, -0.32; 95% CI, -0.58 to -0.07) (Figure 5 and Table 6).

### OMT versus usual obstetric care, sham ultrasound, and untreated for nonspecific low back pain in pregnant women

Three studies with 4 comparisons and 242 participants were included for the analysis of OMT for LBP in pregnant women. Two of these studies showed a significant improvement[48, 53] following OMT, and 1 study[49] showed a non-significant improvement. There was low-quality evidence (downgraded due to inconsistency and imprecision) for a significant difference in favour of OMT for pain (MD, -23.01; 95% CI, -44.13 to -1.88) and functional status (SMD, -0.80; 95% CI, -1.36 to -0.23) (Figures 6 and7, Table 7).

### OMT versus untreated for nonspecific low back pain in postpartum women

Two studies examining OMT for LBP in postpartum women were found, both reporting significant improvement following OMT[54, 55]. There was moderate-quality evidence (downgraded due to imprecision) for a significant difference in favour of OMT for pain (MD, -41.85; 95% CI, -49.43 to -34.27) and functional status (SMD, -1.78; 95% CI, -2.21 to -1.35) (Figures 8 and9, Table 8).

### Adverse events

Of the 15 included studies, only 4 studies reported on adverse events. Two studies reported minor adverse events such as stiffness and tiredness[42, 46]. In the 2013 study, Licciardone et al.[50] reported that 6% of patients had adverse events, but none of the serious events appeared to be related to the treatment intervention, and there were no significant differences between the treatment groups in the frequency of adverse events or serious adverse events. In a personal communication, the authors of another study[48] reported that no adverse events occurred.

## Discussion

To our knowledge, the current review is the first systematic review with meta-analyses examining the effect of osteopathic management for acute and chronic nonspecific LBP based only on studies that used an authentic osteopathic approach. This approach required clinical judgment to individualise the treatment to each patient, rather than applying a single technique or predetermined set of techniques. Because our review had no language or publication restrictions, it is likely the most comprehensive review to date. When included studies were grouped and analysed using meta-analyses, a significant effect for OMT was found for LBP (acute and chronic), chronic LBP, LBP in pregnant women, and LBP in postpartum women. The significant effects were also found to be clinically relevant according to the criteria recommended by the Cochrane Collaboration[38].

The risk of bias in the studies was generally low, with all but 3 of the 15 included studies found to have low risk. None of the studies had a high risk of bias in the randomisation and allocation procedure, but every study had problems with the 3 blinding criteria in the risk of bias assessment. For studies of manual therapy, blinding will usually be an issue because patients tend to be aware of the manual treatment and practitioners cannot be easily blinded from the treatment intervention they deliver. When using methodology assessment tools such as the Risk of Bias instrument, the difficulty of blinding creates a disadvantage for manual therapy studies compared to studies using other interventions like pharmaceuticals which can easily be blinded.

The 2013 study by Licciardone et al.[50] was the largest RCT included in the current review, assessing 455 patients with chronic LBP. The data in the study was not normally distributed and the authors reported medians and interquartile ranges, which were not easily used for meta-analyses. We contacted the authors of this study[50] several times for additional data that could be used in the current analysis, but unfortunately this data was not made available. Subsequently, we needed to transform these data to determine means and standard deviations. We used simulation calculations recommended by Hozo et al.[37], where the median was the best estimator for the mean for sample sizes greater than 60. For the estimation of standard deviations, we calculated average standard deviations based on 3 studies[46, 51, 52], which were similar in outcome, comparison and duration of pain. For the estimation of standard deviations for functional status, we based calculations on two studies[46, 51]. For the margin of error for every estimation, it was possible that a greater difference between our estimation and the real data (i.e., the data was more in favour of the control group) could change our results regarding functional status in chronic and acute and chronic back pain. However, our results for the comparisons were almost identical regardless of whether the transformed data from Licciardone et al.[50] were included or not.

Two previous systematic reviews examined the effect of OMT on LBP. In a 2005 review by Licciardone et al.[28], studies were included if they were performed by an osteopath or osteopathic physician, but the authors also included interventions based on single manual techniques. In the current review, we wanted to examine the effect of studies that used an authentic osteopathic intervention where the clinician was free to use clinical judgment for each patient, as occurs in clinical practice. Consequently, we excluded 2 studies[58, 59] that were included in the 2005 Liccardone et al. review[28] because they involved single techniques. Further, we did not include studies with specific causes of LBP[68]. Although our review did include studies of LBP associated with pregnant and postpartum women, these studies were pooled and analysed separately. Despite these differences, the results and conclusions of our study and of the Licciardone et al.[28] study are similar: both suggested that OMT may be an effective treatment for LBP.

The findings of the current review differ from the results of a recent review by Orrock and Myers[30], largely due to different inclusion criteria. The Orrock and Myers review[30] was restricted to chronic nonspecific LBP and consequently fewer studies met their inclusion criteria. The current review was not restricted to the English language or the published literature, and we located 6 unpublished studies in German[42, 46, 48, 53–55] and 1 study in Italian[52]. Searching the unpublished grey literature for relevant studies is recommended by the Cochrane Collaboration for a more comprehensive search and to avoid publication bias[31]. In addition, the limited number of studies retrieved by Orrock and Myers prevented statistical analysis, whereas we were able to conduct meta-analyses to determine the effect of OMT interventions on LBP. In another systematic review, Posadzki and Ernst[69] examined the effect of osteopathy for musculoskeletal pain. However, Posadzki and Ernst[69] did not specifically address LBP, had only 5 studies that focused on LBP, and had no quantitative analysis, so this study is not comparable to the current review.

OMT appeared to have a larger effect on pain than functional status. Given that our analyses used results from the included studies recorded 3 months after the initial intervention, the subjective experience of pain may be quicker to respond to treatment than function.

It is difficult to assess the relative effectiveness of OMT compared to other specific interventions commonly offered to people with LBP using the available studies. The comparison interventions of the studies included in this review were varied, including sham treatment, usual medical care, physiotherapy, and no treatment; and it was not possible to group and analyse these studies according to the comparison intervention. Rubinstein et al.[32] found that there was high-quality evidence that spinal manipulation, a technique used by osteopaths and other manual therapists, had a small short-term effect on pain, but the effect was not clinically significant. The current review suggested that the effect of OMT was clinically relevant, and it may be that an individualised approach with different techniques contributed to greater effectiveness. However, Rubinstein et al.[32] had access to a greater number of studies with a total of 6070 participants, and the authors were able to examine different time periods for longevity of effectiveness. Walker et al.[70] reviewed studies of chiropractic management of LBP when combined with other interventions, as represents typical practice for many chiropractors, rather than spinal manipulation alone. Chiropractic interventions were found to improve pain in the short and medium term, but not in the long term, compared to other interventions. For functional status, there were short-term improvements, but not in the medium and long term. This review included 12 studies involving 2887 participants, substantially more than the current review on OMT, but only 3 studies had low risk of bias. Given the differing comparison groups in the studies of both reviews, it is not possible to directly compare the effects of OMT and chiropractic management.

Two important limitations of the current review were the sample sizes and comparison groups of the included studies. When studies include few participants and have wide confidence intervals in the analysis, or have small confidence intervals with effects in different directions, heterogeneity is evident and the rating of the quality of evidence should be downgraded according to the GRADE approach recommended by the Cochrane Handbook[31]. Although the majority of the included studies had relatively small sample sizes[39, 42, 46, 48, 51–56], each comparison for chronic and acute pain and for chronic pain contained over 400 participants. However, the comparisons for the conditions of LBP in pregnant and postpartum women contained fewer than 400 participants, which indicated likely imprecision of results and a resultant downgrading of the level of evidence[31]. Future studies with larger samples sizes may change our estimates of effect size for all these comparisons, particularly for LBP in pregnant and postpartum women. There were also a number of different comparison groups in the included studies, including placebo control, usual medical care, and untreated patients.

Considerable heterogeneity was evident in many of the forest plots, which indicated variability and poor overlap in the confidence intervals of the studies. This heterogeneity may be related to the small sample sizes of the studies, as well as the different comparison interventions, which may have had differing effects on pain and functional status. The small sample sizes of many of the studies, the different comparison interventions, and the heterogeneity are limitations of the current review and cause for caution concerning the conclusions. Although we performed meta-analyses on patient groups with different chronicity of symptoms, this did not appear to be a major source of heterogeneity. All patient groups together had a substantial heterogeneity of I^2^ = 85%, but the heterogeneity of only the mixed acute and chronic groups (I^2^ = 81%) and only the chronic groups (I^2^ = 89%) were similar.

It should be noted that the broad widths of the 95% CIs in the forest plots indicate imprecision of the results. This is often the case with systematic reviews of RCTs with small sample sizes. We have interpreted clinical relevance based on the scores of the MD and SMD, but it is necessary to consider lower and higher bounds of the CI and that the true value may lie in this range. The true value could be higher or lower than our point estimator from which we have calculated the clinical relevance, and future studies, using larger samples and robust methodology, may clarify the true point estimate and the clinical effectiveness of OMT for LBP.

The delivery of OMT, which can include a range of manual techniques, is not standardized between practitioners and requires individual clinical judgment for each patient. Most of the included studies provided an indication of the range of manual techniques used for OMT, but the exact interventions performed for each patient were generally unknown. For instance, OMT interventions in the included studies may emphasize different manual treatment approaches. Unfortunately, this lack of specific information from the included studies does not enable us to identify whether responder and non-responder patient groups received different treatments or to understand what the most effective components of OMT interventions are for LBP.

The pain and functional status outcomes analysed in the current review were measured in each study close to 3 months after the initial treatment. Therefore, the longevity of the effect of OMT for LBP cannot be determined, and most of the included studies did not have a longer follow-up period for assessment of pain and functional status. Details about the treatment approach used and clearly reported adverse events should also be included in studies. Future studies should examine the long-term effects of OMT, clearly describe the treatment approach, and report adverse events. Because of the small sample sizes in the majority of the included studies and the heterogeneity in our analyses, these future studies should also have larger sample sizes. Larger RCT studies are expensive to conduct and most of the reviewed studies were unfunded. In order to produce large RCTs examining the effect of OMT on LBP, there must be willingness from osteopathic professional organizations and national funding bodies to support such research.

## Conclusion

To our knowledge, the current systematic review used the most comprehensive search for studies of OMT for nonspecific LBP. The studies we reviewed generally had a low risk of bias, but most had relatively small sample sizes of patients. Our results suggest that OMT improves pain and functional status in acute and chronic nonspecific LBP, in chronic nonspecific LBP, and in pregnant and postpartum women with LBP. Given the small sample sizes, different comparison groups in different studies, heterogeneity, and lack of long-term measurement, larger, high-quality RCTs with robust comparison groups are needed to provide firm conclusions regarding the effectiveness of OMT for LBP.

## Authors’ original submitted files for images

Below are the links to the authors’ original submitted files for images. Authors’ original file for figure 1 Authors’ original file for figure 2 Authors’ original file for figure 3 Authors’ original file for figure 4 Authors’ original file for figure 5 Authors’ original file for figure 6 Authors’ original file for figure 7 Authors’ original file for figure 8 Authors’ original file for figure 9
