# Supplementary figures and images for: Osteopathic manipulative treatment for nonspecific low back pain: a systematic review and meta-analysis
Source: BMC Musculoskelet Disord. 2014 Aug 30;15:286. doi: 10.1186/1471-2474-15-286 (PMC4159549; doi:10.1186/1471-2474-15-286)

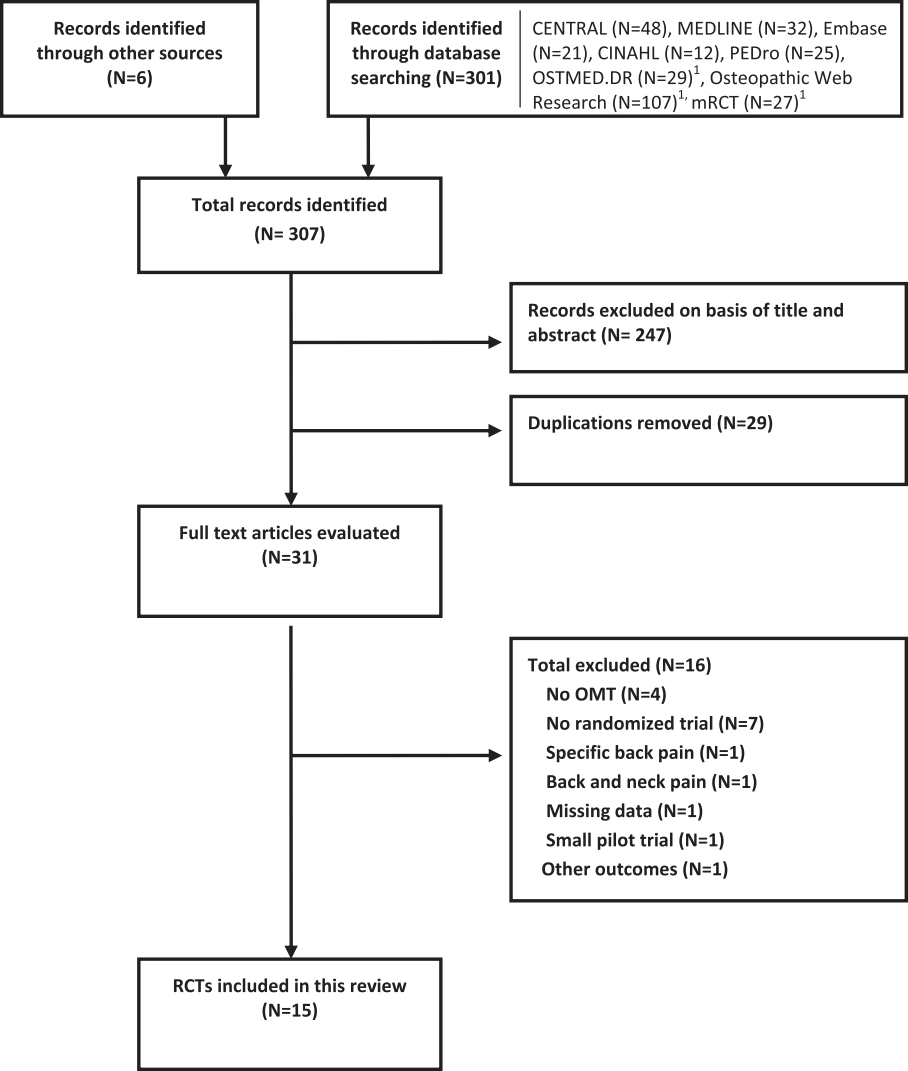

Supplement: Supplementary file 1 — Authors’ original file for figure 1 [file 12891_2014_2231_MOESM1_ESM.pdf]

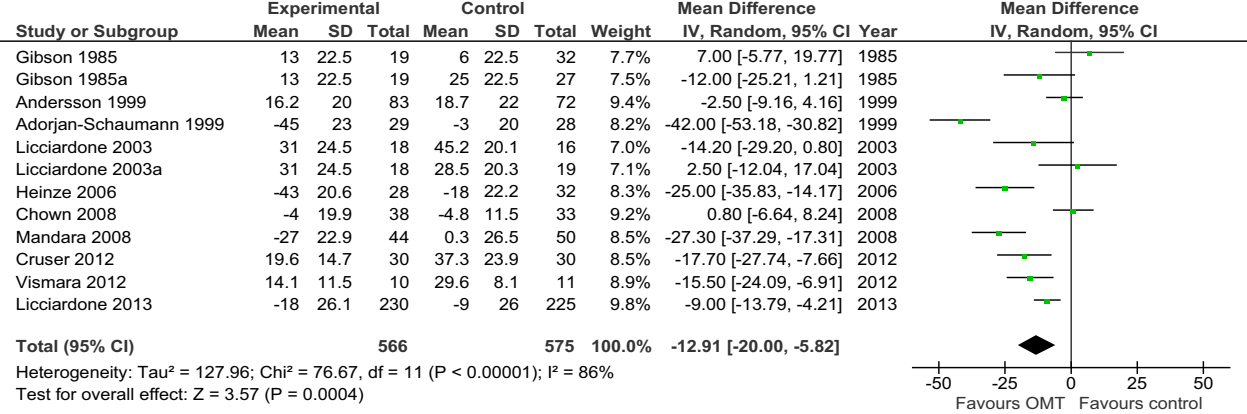

Supplement: Supplementary file 2 — Authors’ original file for figure 2 [file 12891_2014_2231_MOESM2_ESM.pdf]

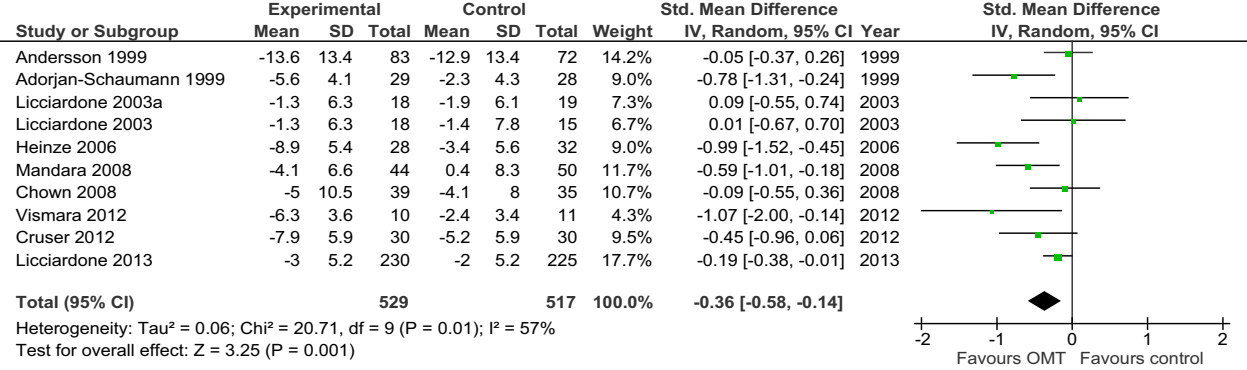

Supplement: Supplementary file 3 — Authors’ original file for figure 3 [file 12891_2014_2231_MOESM3_ESM.pdf]

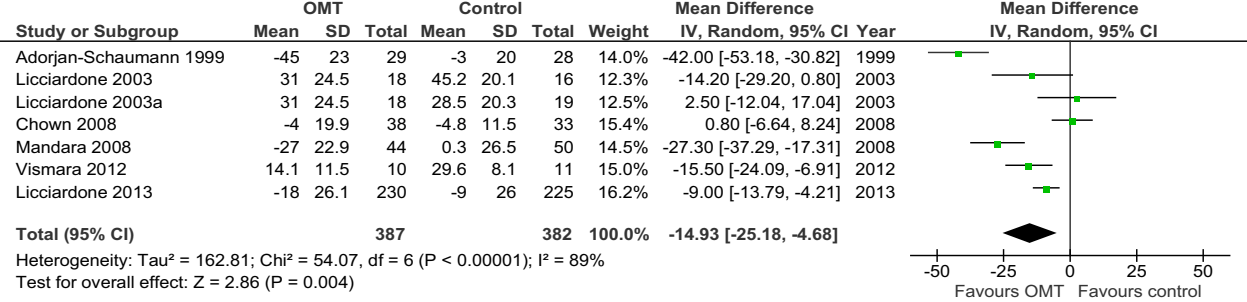

Supplement: Supplementary file 4 — Authors’ original file for figure 4 [file 12891_2014_2231_MOESM4_ESM.pdf]

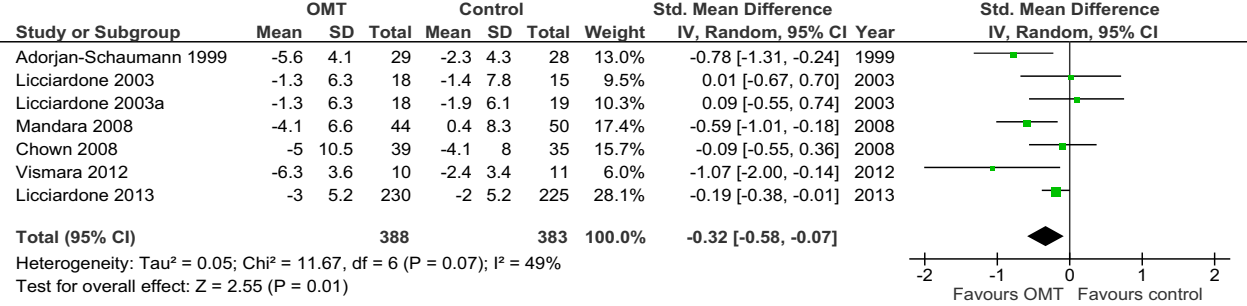

Supplement: Supplementary file 5 — Authors’ original file for figure 5 [file 12891_2014_2231_MOESM5_ESM.pdf]

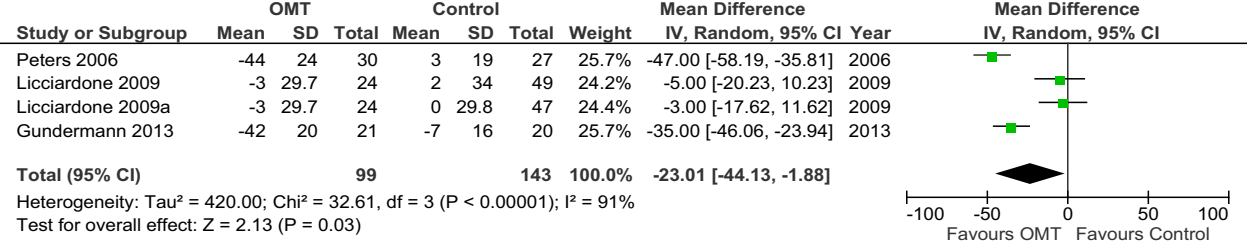

Supplement: Supplementary file 6 — Authors’ original file for figure 6 [file 12891_2014_2231_MOESM6_ESM.pdf]

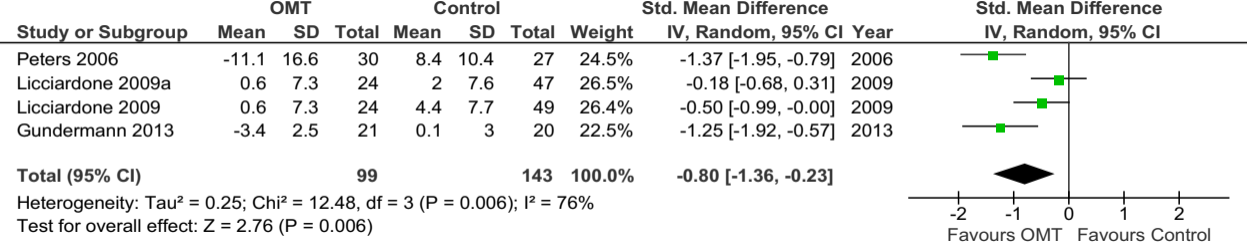

Supplement: Supplementary file 7 — Authors’ original file for figure 7 [file 12891_2014_2231_MOESM7_ESM.pdf]

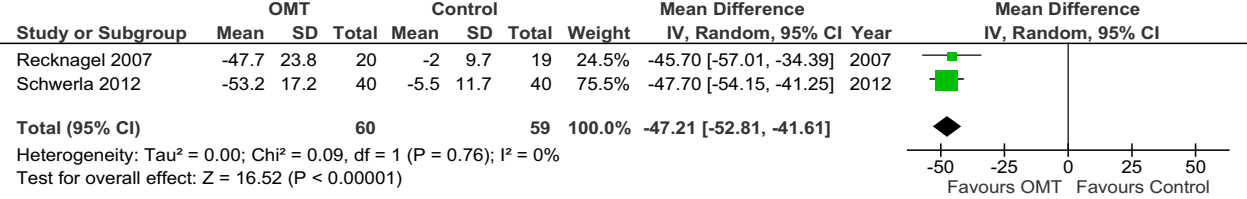

Supplement: Supplementary file 8 — Authors’ original file for figure 8 [file 12891_2014_2231_MOESM8_ESM.pdf]

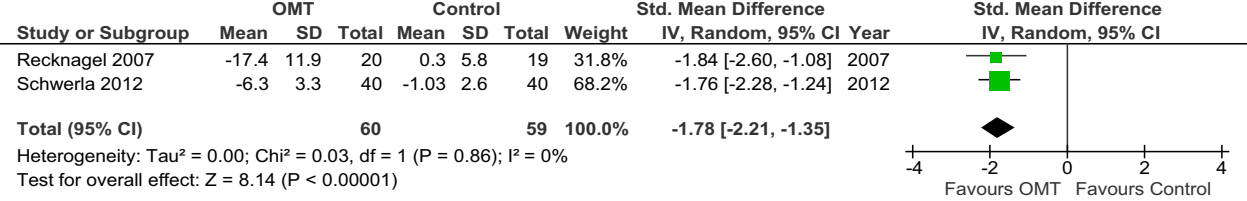

Supplement: Supplementary file 9 — Authors’ original file for figure 9 [file 12891_2014_2231_MOESM9_ESM.pdf]
